# A phase I/II trial of intraoperative breast radiotherapy in an Asian population: 5-year results of local control and cosmetic outcome

**DOI:** 10.1186/s13014-015-0469-6

**Published:** 2015-07-25

**Authors:** Mariko Kawamura, Yoshiyuki Itoh, Masataka Sawaki, Toyone Kikumori, Nobuyuki Tsunoda, Takeshi Kamomae, Seiji Kubota, Tohru Okada, Rie Nakahara, Junji Ito, Hironori Hayashi, Shinji Naganawa

**Affiliations:** Department of Radiology, Nagoya University Graduate School of Medicine, 65 Tsurumai-cho, Shouwa-ku, Nagoya, Aichi 466-8550 Japan; Department of Breast Oncology, Aichi Cancer Center Hospital, Nagoya, Aichi Japan; Department of Breast and Endocrine Surgery, Nagoya University Graduate School of Medicine, Nagoya, Aichi Japan

**Keywords:** Breast cancer, APBI, IORT, Asia, Cosmesis, Recurrences

## Abstract

**Background:**

To date, there are no reports of intraoperative radiotherapy (IORT) use with long-term follow up as a method of accelerated partial breast irradiation (APBI) in Asian countries. We initiated a prospective phase I/II clinical trial of IORT in Japan in 2007, and herein, we report the 5-year follow-up results.

**Materials and methods:**

The following inclusion criteria were used for enrollment in the trial: (1) tumor size < 2.5 cm, (2) desire for breast-conserving surgery, (3) age >50 years, and (4) negative margins after resection. In February 2009, the eligibility criteria were changed to include only patients with sentinel lymph node-negative disease. In phase I, the radiotherapy dose was escalated from 19 Gy/fr to 21 Gy/fr, incremented by 1 Gy per step, with 3 patients in each step. Doses were escalated after all patients in the preceding cohort had completed treatment and exhibited only grade 1 or 2 toxicities at a given dose level. The recommended phase II dose was set at 21 Gy at 90 % isodose. The primary endpoint was early toxicity. Secondary endpoints were long-term efficacy and late toxicity. In addition, Hypertrophic scarring was evaluated retrospectively as a cosmetic outcome by a radiation oncologist.

**Results:**

Between December 2007 and March 2010, 32 women with breast cancer were enrolled in the trial. The median age was 65 years (51–80 years), and the median follow-up time was 6 years. No recurrence or metastasis was observed in any patient. Grade 2 fibrosis was detected in 3 patients as an acute adverse event and in 2 patients as a late adverse event. Ten patients developed a hypertrophic scar 1 year after the IORT; the number of patients decreased to 7 in the 3 years of follow-up.

**Conclusion:**

The first group of female Asian patients tolerated the treatment with IORT in this Phase I/II study and remained recurrence-free for more than 5 years after treatment. However, 24 % of the patients developed hypertrophic scarring, an event that is being further examined in our ongoing multi-center Phase II trial of IORT for early breast cancer.

## Introduction

The standard treatment for early breast cancer is breast-conserving therapy (BCT) with whole-breast external irradiation therapy (WBI) [1]. Local recurrences after BCT with or without WBI arise most often in the same quadrant as the primary cancer [2], which has led to multiple trials on accelerated partial breast irradiation (APBI), many with promising results [3].

Despite the positive results from clinical trials around the world, the Japanese Breast Cancer Society has stated in their guidelines regarding the APBI technique that there is “not enough evidence to perform in clinical use”. In addition, they state, "to start APBI, we need to solve the technical problems arises from breast and body size difference from Western women" [4] because the same target dose would result in a higher skin, heart, and lung dose for patients with smaller breasts, making it difficult to increase the fractional dose.

Intraoperative radiotherapy (IORT) is a form of APBI. The biggest advantage of IORT is its short course of treatment and the fact that adjuvant radiotherapy is performed during surgery, eliminating the need for multiple visits to the hospital for adjuvant radiotherapy. IORT also offers the advantages of excellent delineation of the tumor bed under visual control and a high rate of sparing of the normal tissue, especially the skin [5, 6].

There are multiple IORT techniques currently reported. One technique uses electron-beam accelerators that can be employed in the operating room. This technique was used in the Phase III electron intraoperative radiation (ELIOT) trial by the European Institute of Oncology group [6, 7]. With this method, unnecessary radiation to the heart and lungs can be avoided by placing a shield between the mammary gland and the pectoralis muscle [6]. Another device used for IORT consists of a miniature electron beam-driven X-ray source that provides a point source of low-energy X rays, with a maximum dose of 50 kV. The radiation source is surrounded by a conical sheath with a sphere at the tip of various sizes, and can be inserted into the surgical cavity tumor excision. A multi-center randomized trial using this technique termed “TARGIT” began in March 2000 [5]. The advantage of this technique is that does not involve extensive dissection around the breast to separate it from the skin anteriorly and chest wall posteriorly as is necessary in electron IORT.

Because IORT using electrons can increase a fractional dose without increasing the heart and lung dose for small-breasted patients using a shield [8], we initiated a prospective phase I/II clinical trial of IORT with electrons via a mobile linear accelerator, similar to the ELIOT trial, in Japan in 2007 and reported its safety in a short-term follow up [9]. Here, we report the 5-year follow-up results and a critical evaluation of the study.

## Material and methods

### Study design

The protocol for APBI using the IORT method has been described previously [9]. The study protocol was approved by the institutional ethics committee and was registered with the University Hospital Medical Information Network (UMIN) clinical trial registry, number UMIN 000000918. Written informed consent was obtained from all patients prior to enrollment in the study. The inclusion criteria were as follows: (1) tumor size < 2.5 cm, (2) desire for breast-conserving surgery, (3) age >50 years, (4) negative margins after resection. In February 2009, the eligibility criteria were changed to include only patients with sentinel lymph node-negative disease, due to data supporting node-positive disease as a risk factor for ipsilateral recurrence [1].

For phase I, the radiotherapy dose was escalated from 19 Gy/fr to 21 Gy/fr, incremented by 1 Gy per step. Each cohort comprised 3 patients. Doses were escalated after all patients in the preceding cohort had completed treatment. If patients experienced grade 1 or 2 toxicities at a given dose level, the dose was escalated to the next level. If a patient experienced grade 3 toxicity, dose escalation was discontinued. If a patient experienced grade 4 or 5 toxicities, the study was discontinued. The recommended phase II dose was set at 21 Gy at 90 % isodose.

### IORT procedure

After wide local excision of the primary breast cancer and sentinel node biopsy and/or axillary dissection, a single dose of IORT (19–21 Gy) was delivered via the Mobetron^®^ (Intraop Medical, USA) to the tumor bed using 6 to 12 MeV electron beams. The target area for radiation was at least 2 cm from the margins. Prior to irradiation, an acrylic resin-Cu disc with a diameter of 6–10 cm with 1-cm intervals was inserted between the mammary gland and the pectoralis muscle to shield the heart and lungs from unnecessary dosing. The disc size was chosen to be larger than the applicator size. Patients were excluded from the study if their surgical margins were positive. Adjuvant chemotherapy and endocrine therapy were administered after the surgery if indicated.

### Study assessments

The patients were evaluated every 3 months for the first year after surgery and every 6 months after that for 5 years by several breast surgeons and radiation oncologists independently. Acute and late adverse events including pain, fibrosis, dermatitis, infection, hematoma, and heart and lung events were evaluated prospectively using the National Cancer Institute Common Terminology Criteria for Adverse Events version 3.0. Local recurrence was defined as recurrence or new disease within the treated breast, and was evaluated annually by using mammography and ultrasonography.

Hypertrophic scarring was evaluated retrospectively as a cosmetic outcome by a radiation oncologist using charts or photos. Because the tension to the scar is a critical factor in hypertrophic scarring [10], the rate of hypertrophic scarring was assessed according to the following variables: tumor location, tumor size, and side of the breast.

## Results

Between December 2007 and March 2010, 38 female breast cancer patients were recruited for the trial. Four patients were ineligible for IORT due to positive margins. One patient could not receive IORT due to technical trouble relating to the radiation device. One patient received IORT but was excluded from the evaluation because the pathology indicated a metastatic tumor from lung cancer.

A total of 32 patients were eligible for the evaluation. The median age was 65 years (51–80 years), and the median follow-up time was 6 years (2.5–7 years). Patient characteristics are described in Table 1. Of 32 patients, 2 patients were lost to follow-up at 2.5 years and 3 years, respectively. Neither patient had experienced any adverse events or recurrence at their last follow-up. The other 30 patients completed the protocol-specified 5 years of follow-up. Ipsilateral breast tumor recurrence (IBRT) to either the tumor bed or elsewhere was not observed in any patients. Similarly, regional lymph node or distant metastasis was not observed in any patient. There were no breast cancer-related deaths, but 1 patient died from unrelated causes after 4 years of follow-up. Grade 2 (G2) fibrosis was detected in 3 patients as an acute adverse event and in 2 patients as a late adverse event. No other adverse event greater than G2 including hematoma, infection, pain, or dermatitis was observed. Additionally, no patients experienced any lung or heart events.

**Table 1:** Patients characteristics (*n* = 32)

Characteristics		Number (rate%)
Age	50-59	9 (28 %)
	60-64	6 (19 %)
	65-69	8 (25 %)
	70+	9 (28 %)
Side	Left	14 (44 %)
	Right	18 (56 %)
Tumor site	Upper inner quadrant	12 (38 %)
	Lower inner quadrant	1 (3 %)
	Upper outer quadrant	16 (50 %)
	Lower outer quadrant	2 (6 %)
	Central portion	1 (3 %)
Pathological size	Tis	3 (9 %)
	<1 cm	13 (41 %)
	1-2 cm	15 (47 %)
	>2 cm	1 (3 %)
Positive nodes	None	28 (88 %)
	1	4 (13 %)
Tumor grades	G1, 2	28 (88 %)
	G3	4 (13 %)
Hormone receptor	ER+ and/or PgR+	29 (91 %)
	ER- and PgR-	3 (9 %)
HER2 status	Positive	3 (9 %)
	Negative	29 (91 %)
Molecular subtype	Luminal A	27 (84 %)
	Luminal B	2 (6 %)
	HER2 positive (non-luminal)	1 (3 %)
	Triple negative	2 (6 %)
ASTRO consensus statement categories for the application of APBI	suitable	16 (50 %)
	cautionary	12 (38 %)
	unsuitable	4 (13 %)
Adjuvant systemic treatment	None	5 (16 %)
	Endocrine therapy	22 (69 %)
	Chemotherapy	3 (9 %)
	Endocrine and Chemotherapy	2 (6 %)

**Fig. 1:**
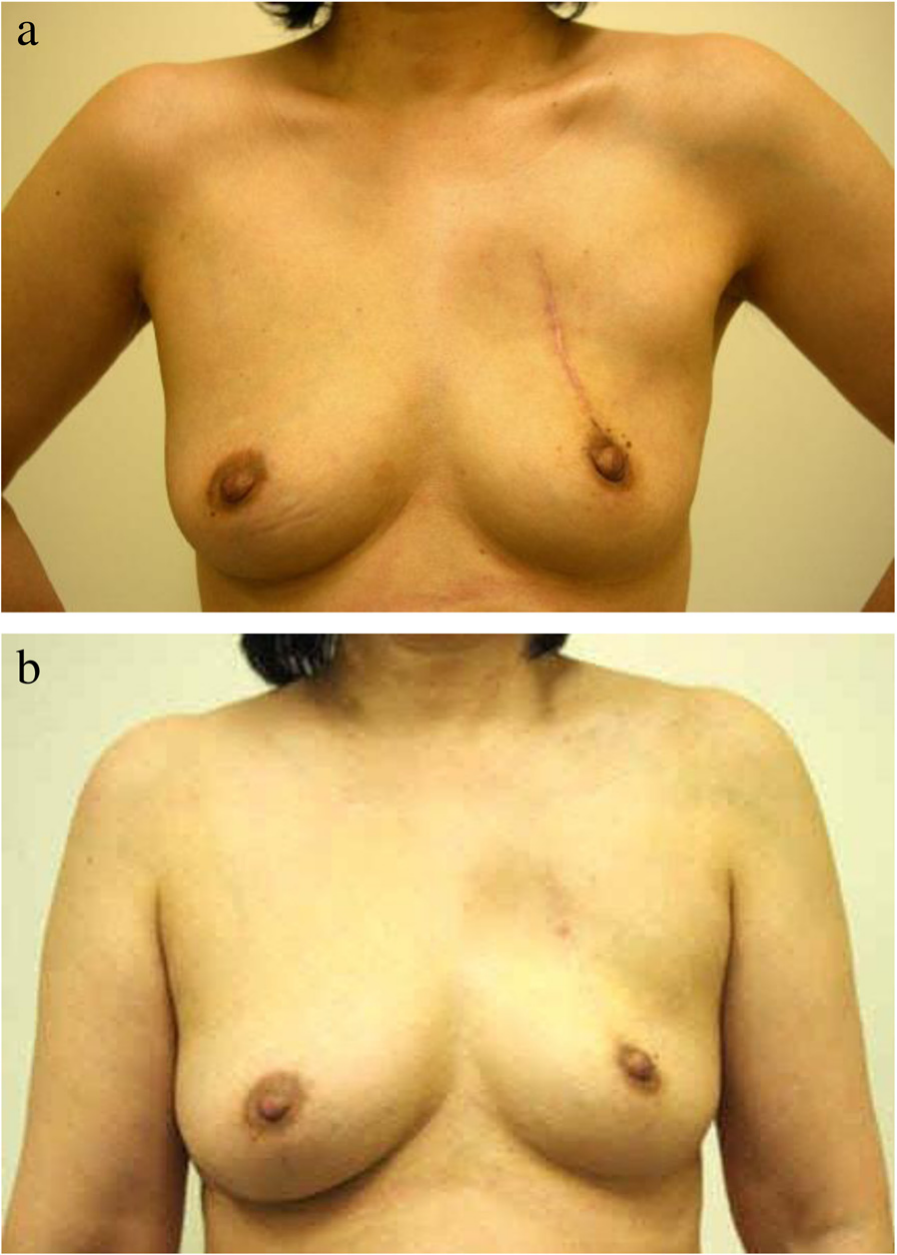
A patient with hypertrophic scarring that disappeared by the 3-year follow-up. **a**) The scar 1 year after IORT. **b**) Hypertrophic scarring at the 3-year follow up

**Fig. 2:**
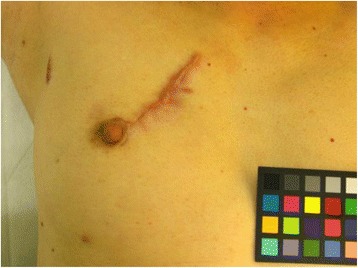
Hypertrophic scarring or keloid lasting for more than 3 years

Of 32 patients, we were unable to evaluate cosmesis in 3. In the other 29 patients, hypertrophic scarring was observed in 10 patients 1 year after the IORT; the number of patients exhibiting hypertrophic scarring decreased to 7 patients in the 3-year follow-up (Fig. 1) without any treatment for hypertrophic scarring. Table 2 outlines the potential predictors of hypertrophic scarring lasting for more than 3 years, and Fig. 2 depicts hypertrophic scarring lasting for more than 3 years. Because of the small number of patients, a valid statistical evaluation was not possible.

**Table 2:** Predictors of hypertrophic scar lasted for more than 3 years

		Rate of hypertrophic scar
Scar location	Inner (AB)	2/13(15 %)
	Outer (CD)	5/19(26 %)
Sides	Right	6/18(33 %)
	Left	1/14(7.1 %)
T size	<1 cm	4/16(25 %)
	≧1 cm	3/16(19 %)

## Discussion

This study is the first IORT report in Asian women with long-term follow-up and cosmetic evaluation focused on hypertrophic scarring. Importantly, our study did not report any grade 3 or greater adverse events as described by other previous studies [7, 11].

Veronesi et al. reported the 5.8-year follow-up results of a randomized controlled equivalence trial comparing IORT with electrons and external radiotherapy (EBRT) for early breast cancer (ELIOT) in 1305 patients [7]. They concluded that APBI using the IORT technique resulted in a significantly higher IBRT of 4.4 % compared to 0.4 % in the whole-breast irradiation group. However, the differences in survival were not reported and the adverse events to the skin were significantly fewer in the IORT group [7]. In addition, it is very important to note that the tumor recurrence rate decreases to 1.5 % if patients are selected based on their suitability for APBI [12]. Vaidya et al. reported similar results from the TARGIT-A trial, that the IBRT rate was higher, but that there were fewer non-breast cancer deaths with TARGIT, attributable to fewer deaths from cardiovascular causes and other cancers. They also reported that grade 3 or 4 skin adverse events were significantly reduced in the IORT group (4 of 1720 vs. 13 of 1731, *p* = 0.029) [11]. These two large randomized studies concluded that IORT is a considerable option for selected breast cancer patients.

Trials such as the Prime II and CALGB9343 trial [13, 14] reported that local relapse rates for cohorts treated without whole-breast radiation therapy in elderly low-risk patients were acceptable compared to those using radiotherapy. In addition, those studies have shown that these differences in local relapse did not result in a difference in axillary recurrence, distant metastasis, or breast cancer-specific survival.

Table 3 summarizes the IBRT rate reported from the randomized IORT study and omission of radiation therapy for elderly patients. Although it is difficult to compare the results of different studies, the recurrence rates are very similar in the IORT and omission of radiotherapy groups even though the omission group has favorable prognostic factors. As IORT can be administered during surgery, avoiding unnecessary irradiation to the heart, lungs, and skin, a randomized control study comparing complete omission of radiotherapy and IORT should be conducted before evaluating complete omission of radiotherapy alone because recurrence leading to mastectomy would lower the patient's quality of life.

**Table 3:** Studies of the IORT and the role of irradiation for elderly

study	No. of patients	Follow-up (years)	Age	Treatment	Local Recurrence rate	*P*
ELIOT [7]	1305	5.8	48-75	IORT	4.4 %	<.0001
				WBI	0.4 %	
TARGIT-A [11]	1222	5	>45	IORT	3.3 %	.042
				WBI	1.3 %	
PRIME II [13]	1326	5	>65	Tam or AI	4.1 %	.0002
				Tam or AI + RT	1.3 %	
CALGB9343 [14]	636	12.6	>70	Tam	10 %	<.001
				TamRT	2 %	

*p*, statistical significance; *IORT*, intraoperative radiotherapy; *WBI*, whole breast irradiation; *Tam*, tamoxifen; *AI*, aromatase inhibitor; *RT*, radiotherapy; *TamRT*, tamoxifen plus radiation therapy

In our study, we did not detect tumor recurrence in any patient. We included 4 patients with one lymph node metastasis before February 2009. All 4 patients underwent axillary dissection of level I and II lymph nodes and additional systemic therapy; 3 patients were administered adjuvant chemotherapy and 1 patient received hormonal therapy. We also included a cautionary group based on the ASTRO consensus statement [15] (Table 1). All but one cautionary group patient received additional adjuvant systemic therapy. We believe this additional adjuvant therapy played an important role in our reported lack of tumor recurrence.

The biggest advantage of IORT from the patient’s perspective is that adjuvant radiotherapy is performed during tumor resection, and therefore, recurring visits for radiotherapy are unnecessary. Other types of APBI, such as brachytherapy and three-dimensional conformal radiotherapy can also shorten the duration of radiotherapy, but they do not approach the timeframe of IORT. However, the biggest advantage of IORT from the medical perspective may be different from that of the patient. Patients who receive IORT with electrons and thoracic shield will not undergo unnecessary irradiation to the heart, lungs, and skin. This is very important, especially in the Asian population whose average breast size is smaller than that of the Western population, thereby resulting in increased relative radiotherapy doses being received by healthy structures such as the heart, lungs, and skin. As reported in our results, we did not observe any heart, lungs, or skin toxicities, suggesting that IORT may be a suitable APBI technique for a small-breasted population.

The major disadvantage of IORT may be the fact that 7/29 patients experienced hypertrophic scarring that resulted in a poor cosmetic outcome. This finding has never been reported elsewhere. Hypertrophic scarring is known to occur more commonly in Asian or other non-Caucasian populations [16], so this finding maybe very important to note when applying IORT to these populations. Although we do not have data regarding the rate of hypertrophic scarring after mastectomy or BCT without irradiation, in our institution, only 6 % (5/81 patients) of the breast cancer patients treated in 2010 with BCT and EBRT experienced hypertrophic scarring. By comparison, the rate of hypertrophic scarring for patients treated with IORT was higher. As IORT requires a wider excision to insert the shield disk, patients without a risk of hypertrophic scarring should be carefully chosen. Although we failed to prove statistical significance in identifying potential predictors of hypertrophic scarring, evaluation of the effect of tumor location on the occurrence of hypertrophic scars is of great interest. As mentioned previously, tension to the scar is a critical risk factor for hypertrophic scarring and that hand domain and incision type have an intimate involvement in the tension to the scar. We are now conducting an ongoing multi-center phase II trial of IORT using electrons, which we believe will answer this question. However, if the risk of hypertrophic scarring is known prior to the surgery, it may be safer to choose conventional whole-breast radiotherapy, which produces a smaller scar than IORT, and irradiation to that scar may suppress the occurrence of hypertrophic scarring [16].

The major limitation of our study was its small number of patients. This phase I/II study included only 32 patients; a larger population is necessary to evaluate if this method is suitable for Japanese or other Asian populations. However, with regard to the potential use of APBI in the Asian population, it is very important to report its safety, including both early and late toxicities, and ensure that 21 Gy of electron beams to the tumor bed is safe for the Japanese population as demonstrated in the Eliot trial [7].

## Conclusion

The first group of female Asian patients tolerated the treatment with IORT in this Phase I/II study and remained recurrence-free even after more than 5 years of follow-up. However, hypertrophic scarring was identified in 24 % of the patients and will be further investigated in our ongoing multi-center Phase II trial of IORT for early breast cancer.

## Abbreviations

APBI: Accelerated partial breast irradiation
ASTRO: American society for radiation oncology
BCT: Breast-conserving therapy
EBRT: External beam radiation therapy
G2: Grade 2
IORT: Intraoperative radiotherapy
UMIN: University Hospital Medical Information Network
